# Physiological aging and life-cycle labor supply across countries

**DOI:** 10.1371/journal.pone.0294952

**Published:** 2023-11-29

**Authors:** Casper Worm Hansen, Carl-Johan Dalgaard, Holger Strulik

**Affiliations:** 1 Department of Economics, University of Copenhagen, Copenhagen, Denmark; 2 Department of Economics, University of Göttingen, Göttingen, Germany; Universität Hamburg: Universitat Hamburg, GERMANY

## Abstract

We construct a cohort-based frailty index for 180 countries over the period 1990-2019. We use this measure of physiological aging to estimate the impact of deteriorating health on labor force participation. Our three-dimensional panel framework, in which the unit of observation is a cohort in a given country at a given age, allows us to control for a range of unobserved factors. Our identification strategy further exploits a compensating law of physiological aging to account for reverse causality. We find a negative effect of physiological aging on labor market participation: an increase of the frailty index by one percent leads to a reduction of labor force participation of about 0.6 (±0.2) percentage points. Since health deficits (in the frailty index) are accumulated at a rate of about 3 percent per year of life, almost all of the age-related decline in labor force participation can be motivated by deteriorating health.

## 1. Introduction

The world is aging. This fact is an inevitable consequence of the demographic transition that has been sweeping most countries in the world since the 19th century and continuing throughout the 20th century with falling fertility and mortality rates [1]. The process of population aging has later been fueled by a longevity transition in which mortality rates at later ages have been falling as well [2]. It has been argued that population aging is an important factor in economic development and growth, particularly through its impact on labor supply and productivity [3]. The idea is that labor force participation rates follow a hump-shaped path over the life cycle, typically peaking in the 40s, and so if more people reach later ages, aging is mechanically reducing the aggregate size of the labor force.

This line of reasoning, however, implicitly assumes that labor force participation depends on chronological age and is not influenced by factors of physiologically aging that are naturally embedded in population aging, such as declining functionality of the human body. For example, the popular Mincer–wage equation [4] postulates that the logarithm of wages depends positively on age and negatively on age-squared. According to this approach, increasing chronological age ultimately leads to a decline in wages and to individuals retiring. The quadratic age term is thought to represent both the effect of experience and the effect of declining physiological health, which at some point overtakes the positive effect of experience. This simplification, however, has important policy implications, since chronological aging inevitably advances by one year each year, while physiological age (the state of health) is malleable. A refined Mincer equation that explicitly accounts for health can capture the feature that individuals of a given age change their labor supply as their health improves or deteriorates.

A distinction between chronological age and physiological age is particularly relevant when there is a trend towards improving health for a given age, such that physiological aging is slowed down and possibly eventually abandoned [5–8]. Physiological aging can be explained by increasing loss of redundancy in the human body and therewith deteriorating reliability and increasing frailty [9, 10]. According to this view, the increasing withdrawal of workers from the labor market is caused by declining productivity due to deteriorating muscle strength and motor skills, musculoskeletal pain, and decreasing cognitive abilities [11–15].

There exists a microeconomic literature that estimates the impact of health on labor supply using individual data. Depending on empirical approach and health measurements, results differ from study to study (see [16] for a review). The recent study by Blundell et al. use alternative health measures, controls for individual fixed effects and initial conditions, and estimates that deteriorating health explains up to 15% of the decline in employment between ages 50 and 70 in England and the US [17]. Vandenberghe estimates the association between health and employment at age 50 (and other labor market indicators) for 20 European countries and uses the estimates for counterfactual predictions of employment at age 70 [18]. He finds that deteriorating health explains at most 35 percent of the observed reduction in employment.

Another literature uses calibrated models of the individual life cycle to asses how health shocks affect labor supply and other life cycle choices [19–21]. This approach allows to identify several channels, aside from declining productivity, through which health could affect labor supply, such as utility gains from leisure, indirect effects through the impact of increasing life expectancy on savings and education decisions; disability benefits, the pension- and health-insurance system, and medical expenses. Capatina estimates that the removal of health shocks leads to an increase of labor supply of non-college educated individuals of 10.8 percent and that the largest part (7.4 percent) operates through changing labor productivity [19]. A related literature uses life cycle models to explain how technological progress and perpetual wage growth contributed to the continuous rise of the length of the retirement period over the last century [22, 23]. An extensive literature investigates how health affects the retirement decision through life expectancy and mortality [15, 24–29].

In this paper, we propose an alternative approach to study at the aggregate level of countries how physiological aging influences age-specific labor force participation rates. In order to do this, we draw on research in the fields of biology and medicine to compute an empirical measure of physiological aging for most countries in the world from 1990 to 2019. This micro-founded variable, known as the frailty index, aggregates age-specific prevalence rates of 32 age-related diseases to a cohort-, age-, country-, and gender-specific measure of aging. The frailty index has been developed by Mitnitski et al. [30, 31] and is an established method to assess human aging, used by hundreds of studies in gerontology and medical science. The index measures the proportion of a large number of age-related health conditions that an individual has. It has been established that it plays no role whether specific health deficits are included in the index, as long as there are enough of them included [32]. This property is due to the fact that health deficits are interconnected. For example, high blood pressure is associated with heart disease, congestive heart failure, vision, and memory loss. The index therewith records in a single number the process of biological aging defined as the “intrinsic, cumulative, progressive, and deleterious loss of function” [9].

The frailty index’s quality has been established by its predictive accuracy for mortality, as well as for other health outcomes such as the likelihood of nursing home institutionalization and becoming a disability insurance recipient. [33–35]. Dalgaard and Strulik integrated the frailty index into an economic life cycle theory of health, aging, and death and provided a biologically founded framework to discuss health behavior and health outcomes [36]. Applications consider, for example, the gender gap in mortality [37], the health gain from marriage [38], fetal origins of late-life health [39], and particular health behavior such as addiction [40], self-control problems [41], and adaptation to poor health [42].

Most of the literature on the frailty index considers aging of individuals. Here, we built on the study of Dalgaard et al. [43] who used data from the Global Burden of Disease (GBD) study [44] to construct a frailty index of populations. In a panel analysis, controlling for country- and time fixed effects, Dalgaard et al. showed that the population-level frailty index reflects a number of regularities previously found at the individual level [43]. Specifically, it was shown that the frailty index increases with age in exponential fashion, at a rate of 2.8 to 3.0 percent per year of age. This speed of aging was found to be very similar across countries from different continents and at different levels of aggregate income. Moreover, for a subset of countries, for which mortality data was available, the study showed a strong association of health deficits and mortality. It found that a one percent rise in the frailty index was associated with an approximately three percent rise in the mortality rate.

We extend this research by constructing the frailty index for cohorts. We then combine the frailty index with cohort-, age-, country-, and gender-specific labor force participation rates to estimate a cohort-based life-cycle model of labor supply for 180 countries over the period 1990–2019. The comparison of cohorts within the same country over the life cycle allows us to exploit interaction fixed effects to control for a range of unobserved factors (e.g., cohort-gender-country fixed effects).

While our higher dimensional panel model allow us to hold a range of unobserved factors constant, the problem of reverse causality makes it difficult to estimate the effects of physiological aging on labor market participation. In a series of studies, Marmot argues that work affects health due to occupational stress, social position, and sense of being in control of one’s life [45, 46]. Another strand of literature argues that health status is negatively affected by blue collar and physical job burden [47–49]. Specifically, in the context of the frailty index, exposure to physical or psychosocial job burden as well as employment in blue collar occupations has found to be associated with a faster accumulation of health deficits during the work life [50]. In order to address the issue of reverse causality, our identification strategy leverages a compensating regularity of the frailty index, implying that there exists a strong negative relationship between initial health deficits, measured at the beginning of working life, and the rate of health deficit accumulation. This pattern in the frailty index has been documented in previous research for samples of individuals [5, 6, 31] and it is also present in our global country study. In other words, this means that the level of deficits at the beginning of working life is predictive of the (log) change in deficits at each age.

We find that physiological aging has a negative effect on labor force participation rates. Our baseline estimate suggests that, if the frailty index increases by one percent, the labor force participation rate decreases by 0.25 percentage points. In 2SLS estimations, the point estimates increases in absolute value to more than 0.6 percentage points. Noticing that the frailty index increases, on average, by 2.6 to 3.0 percent per year over the life cycle, these estimates indicate that there is a substantial drag on labor supply of deteriorating health due to physiological aging.

Our results provide an empirical reason as to why the relationship between age and labor force participation eventually becomes negative. When health deficits are missing in regressions of labor markets participation, the age coefficients suggest that the positive effect of chronological age (experience) on labor supply is reverted around age 35–39. When we control for health deficits, chronological age exerts a positive impact until age 55 and chronological age alone would not be able to explain deteriorating participation rates. The decline by almost 30 percentage points from ages 30–40 to ages 60–64 is almost fully accounted for by deteriorating health.

The paper proceeds as follows. In the next section, we introduce the frailty index and its measurement at the macro level, explain the compensation law of morbidity, and set up a simple life cycle model to derive how physiological aging affects labor force participation. In Section 3, we introduce our data and in Section 4 we explain our estimation strategy. In Section 5 we present the results. Section 6 discusses implications and concludes.

## 2. Measurement and theory

### 2.1 The frailty index

For individuals, the frailty index is constructed as the proportion of the total potential deficits (**a**ilments) *a* = 1, …, *A* present in an individual. Specifically, for individual *j* with gender *g* living in country *i* the frailty index is:

$$\begin{array}{c} d_{jgi}=\frac{1}{A}\sum_{a=1}^{A}1_{jgi}(a). \end{array}$$ (1)

The indicator function**1**_*ji*_ (*a*) takes the value 1 if individual *j* suffers from deficit *a*. The criteria for the selection of health deficits are outlined in [32]: they need to be aging-related (prevalence increasing in age), associated with health status, not saturate too early, and cover a broad range of deficits. The results are robust to the inclusion or omission of specific deficits, provided that a sufficiently large number of deficits (30 to 40) are included in the index [32, 51]. As explained in the Introduction, the intuition for the remarkable feature that the appearance of specific deficits is not decisive lies in the micro-foundation of the frailty index in reliability theory [10] and in a network theory of human aging [52]; theories that emphasize that health deficits are connected. The large literature of micro studies using the frailty index typically uses an unweighted index because the weighting of the items limits generalizability across populations and studies. Studies comparing weighted and unweighted indices found that weighting improves prediction quality (for mortality) slightly, but that these gains are not large enough to give up generalizability [53].

In light of its simplicity and intuitive nature, it is perhaps unsurprising that the frailty index has been applied in hundreds of studies until now. However, in most of these studies the index is computed for samples of individuals. Here, we follow the methodology developed in [43] and compute the frailty index for populations. Specifically, based on the individual frailty index from Eq (1), the average frailty index of cohort *c*, gender *g*, in country *i* is:

$$\begin{array}{c} D_{cgi}=\frac{1}{P_{cgi}}\sum_{j}^{P_{cgi}}d_{jgi}, \end{array}$$ (2)

where *P*_*cgi*_ is the number of individuals belonging to cohort *c*, gender *g*, living in country *i*. Inserting Eq (1) and rearranging, allows us to write Eq (2) as:

$$\begin{array}{c} D_{cgi}=\frac{1}{A}\sum_{a=1}^{A}\frac{P_{acgi}}{P_{cgi}}, \end{array}$$ (3)

where *P*_*acgi*_/*P*_*cgi*_ is the prevalence rate of age-related (disease) condition *a* in cohort *c*, gender *g*, country *i*. Therefore, in order to work out the aggregate frailty index for this particular age cohort, we simply need to calculate the average of *A* prevalence rates, *P*_*acgi*_/*P*_*cgi*_. These prevalence rates are available for most countries in the world in the GBD database [44].

### 2.2 Compensation law of deficit accumulation

Research on the dynamics of health deficits accumulation has established that health deficits grow, on average, at a constant rate with advancing age [5, 31, 54]. Exponential growth means that the logarithm of health deficits increases linearly in age such that a person *j* at age *t* displays *D*_*j*_(*t*) health deficits:

$$\begin{array}{c} D_{j}\left(t\right)=D_{j}\left(0\right){\text{e}}^{\mu_{j}t}\;\Leftrightarrow \;\text{log}\;D_{j}\left(t\right)=\text{log}\;D_{j}\left(0\right)+\mu_{j}t. \end{array}$$ (4)

The exponential growth of the frailty index is akin to the exponential growth of the mortality rate, known as Gompertz law, one of the most important quantitative tools in demography, gerontology, and in the analysis of failure times for living organisms [55, 56]. The joint exponential increase in mortality and the frailty index reflects the fact that people do not die because of their chronological age but because of their accumulated health deficits and functional limitations.

Reliability theory provides an explanation why systems (such as bodies or organs) are aging, i.e. display failure rates that are increasing with age, although they consist at the lowest level of non-aging elements with a constant failure rate. The main insight is that complex system consist of redundant elements and that, as redundant elements expire at a constant failure rate, the failure rate of the complex system increases with age of the system due to the decreasing redundancy. The specific architecture of redundancy in complex systems then explains why mortality and frailty increase exponentially with advancing age, i.e. at a constant rate (for a detailed exposition, see [10, 57]). Physiological aging of humans is thus explained as loss of redundancy over time. For example, for young adults, the functional capacity of human organs has been estimated to be tenfold higher than needed for survival [58]. A meta for 13 human organ systems computed a mean loss of functional capacity of 0.65 percent per year [59].

Reliability theory can also be used to explain another generality in gerontology, the compensation effect of mortality, also known as Strehler-Mildvan correlation: in sub-populations where the initial mortality rate is high the acceleration of mortality with advancing age is low. The observed inverse log-linear relationship between initial mortality and increase of mortality implies the convergence of mortality rates and the existence of a common age at which mortality of the sub-population coincide [10, 60]. A similar compensation has been found for health deficits: in sub-populations where initial health deficits are high, the rate of health deficit accumulation is low such that a common age exists at which the sub-populations display the same frailty index. This feature has been shown for populations from different countries as well as within countries for populations of men and women. [5, 31]. Women, on average display more initial deficits but develop further health deficits more slowly. According to reliability theory, sub-populations with lower initial frailty index (i.e. greater redundancy) are predicted to display a faster increase of the frailty index (failure rate of organs and body systems) with advancing chronological age, which explains the compensation effect.

The convergence of health deficits predicted by the compensation effect may seem puzzling at first glance given the often observed divergence in health at the individual level. For example, it has been found that health and the frailty index of blue collar workers and white collar workers diverge with advancing age [50, 61]. The key insight here is that the convergence result applies to differences in *initial deficits* in samples of *populations* (for example, stratified by countries or by gender) while the divergence result is observed for differences in *persistent exposure* to health conditions in samples of *individuals*.

**Fig 1:**
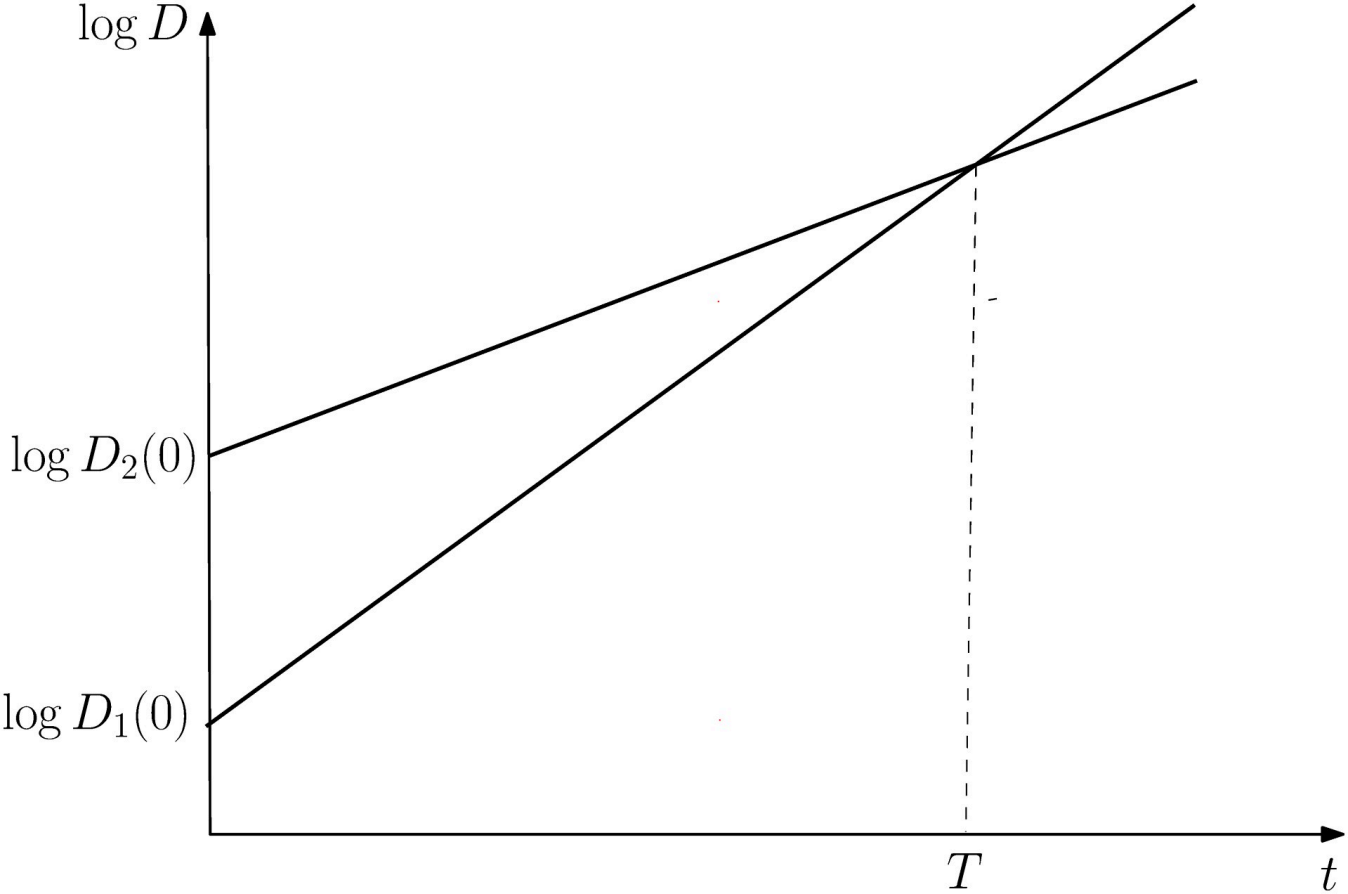
Compensation law of health deficits.

Formally, the compensation effect means that there exists an age *t* = *T* at which all individuals from a population are predicted to display the same frailty index, *D*_*j*_(*T*) = *D*(*T*) for all *j*. Fig 1 displays the compensation law for two sub-populations *j* = 1, 2. Sub-population 1 starts with fewer health deficits but ages faster such that at age *T* both sub-populations display the same frailty index. Formally the compensation law states that

$$\begin{array}{c} \text{log}\;D_{j}\left(0\right)=\lambda -\mu_{j}T. \end{array}$$ (5)

The parameters *T* and λ ≡ log *D*(*T*) have been estimated with great precision and the estimates suggest that *T* is about 95 to 105 years, depending on the populations investigated [5, 6, 31].

Substituting *D*_*j*_(0) in (4) by (5), we obtain:

$$\begin{array}{c} \text{log}\;D_{j}\left(t\right)=\lambda -\mu_{j}\left(T-t\right). \end{array}$$ (6)

Recall, that due to (4), initially healthier individuals (with fewer health deficits) display a higher rate of aging *μ*_*j*_ such that, for any *t* < *T*, they display fewer health deficits *D*_*j*_(*t*) than initially less healthy individuals, see Fig 1.

We will exploit the compensation law in our empirical strategy. In particular, taking age differences of (4), we obtain Δ log *D*_*j*_ = *μ*_*j*_ and inserting (5) we obtain the association between the growth rate of deficits and the initial level of deficits, Δ log *D*_*j*_ ≡ log *D*_*j*_(*t*) − log *D*_*j*_(*t* − 1) = (λ/*T*) − (1/*T*) log *D*_*j*_(0). Considering individuals *j* drawn from different countries *i*, cohorts *c*, and gender *g*, and allowing for country- cohort, and gender-fixed-effect *FE*, we obtain:

$$\begin{array}{c} \Delta \;\text{log}\;D_{cgi}=\alpha \;\text{log}\;D_{cgi}\left(0\right)+FE, \end{array}$$ (7)

in which *α* = −1/*T*, Δ log *D*_*cgi*_ = *μ*_*cgi*_ is the growth rate of health deficits and *D*_*cgi*_(0) are initial health deficits of an individual from cohort *c*, gender *g*, and country *i*. Eq (7) is our theoretical motivation for the first-stage regression given in Eq (14) below.

### 2.3. Labor force participation

In this section, we integrate health deficits in a standard model of optimal labor force participation and derive the structural model for the regression analysis. Consider an individual *j* experiencing instantaneous utility *u*(*c*_*j*_) − *ϕ*_*j*_*ℓ*_*j*_, in which *c*_*j*_ is consumption, *ℓ*_*j*_ is labor supply and *ϕ*_*j*_ is disutility from work. The utility function *u* exhibits positive and declining marginal utility *u*′ > 0, *u*″ < 0. Here we focus on labor supply at the extensive margin such that *ℓ*_*j*_ ∈ {0, 1}, i.e. individuals are in or out of the labor force. Individuals maximizes lifetime utility:

$$\begin{array}{c} \int_{0}^{T}[u\left(c_{j}\left(t\right)\right)-\phi_{j}\ell_{j}\left(t\right)]{\text{e}}^{-\rho t}dt, \end{array}$$ (8)

with time preference rate *ρ*, subject to the budget constraint ${\dot{k}}_{j}\left(t\right)=rk_{j}\left(t\right)+1_{\ell_{j}\left(t\right)=1}w_{j}\left(t\right)-c_{j}\left(t\right)$, in which *t* is age, *k*_*j*_ are assets, *r* is the interest rate, *w*_*j*_ is the wage rate and $1_{\ell_{j}=1}$ is an indicator function that assumes the value of one if the individual is in the labor force (and zero otherwise). Parameters depend potentially on gender-, cohort-, and country-specific characteristics. When possible without loss of information, the respective indices are suppressed to avoid notational clutter.

The wage at age *t* is a function of age and health. Let initial age, i.e. the age at which individuals enter the workforce be normalized to zero. The wage-per-age function is then given by:

$$\begin{array}{c} w_{j}\left(t\right)=\omega_{j}e_{j}\left(t\right)g\left(D_{j}\left(t\right)\right), \end{array}$$ (9)

in which the initial wage *ω*_*j*_ summarizes education and other parameters given at the point of entry in the workforce. Allowing for pension income, the left-hand side of (9) would be rewritten as *w*_*j*_(*t*)(1 − *ξ*(*t*)), in which *ξ*(*t*) is the replacement rate. The structure of the problem would be preserved. The term *e*_*j*_(*t*) captures experience, which grows with increasing age (duration of stay in the workforce). As discussed in the Introduction, according to the original Mincer-wage equation, a negative term of “experience squared” causes wages to decline in old age such that individuals withdraw from the labor force. Here, we consider instead that productivity is reduced by the presence of health deficits, *g*(*D*). The function *g* is declining in deficits and concave such that *g*′ < 0 and *g*″ ≤ 0. Experience is assumed to be a positive and concave function of age with $e_{j}^{\prime }>0$ and ${\text{lim}}_{t\to \infty }e_{j}^{\prime }=0$. This means that, eventually, with increasing age, health deficits become the dominating force on productivity, which causes individuals to exit the labor force.

Individuals maximize (8) subject to (9) and (10), the asset accumulation function and potentially other dynamic constraints. Irrespective of the complexity of the underlying dynamic problem, the first order condition for labor supply is straightforward since it does not reflect intertemporal trade-offs but the intra-temporal trade-off between working and not working. Specifically, the first order condition for labor supply reads:

$$\begin{array}{c} \phi_{j}\leq u^{\prime }\left(c_{j}\left(t\right)\right)\omega_{j}e_{j}\left(t\right)g\left(D_{j}\left(t\right)\right), \end{array}$$ (10)

which requires that the marginal disutility from work is not larger than the marginal utility from work, consisting of earned income evaluated at the marginal utility *u*′(*c*) that a unit of income can buy. At the point of optimal retirement, the participation constraint (10) holds with equality. Inspection of (10) shows that, ceteris paribus, i.e. for given parameters and functional forms, individuals who have accumulated more health deficits withdraw earlier (at smaller *t*) from labor force participation.

Alternatively, we could arrive at a condition isomorph to (10) by assuming that health deficits leave productivity unaffected but increase the disutility from work, such that *ϕ*_*j*_ is replaced by *ϕ*_*j*_*f*(*D*) with *f*′ > 0, *f*″ ≥ 0. In reality, both mechanisms are likely operative and we can imagine that one function *g*(*D*) captures their joint effect in reduced-form.

**Fig 2:**
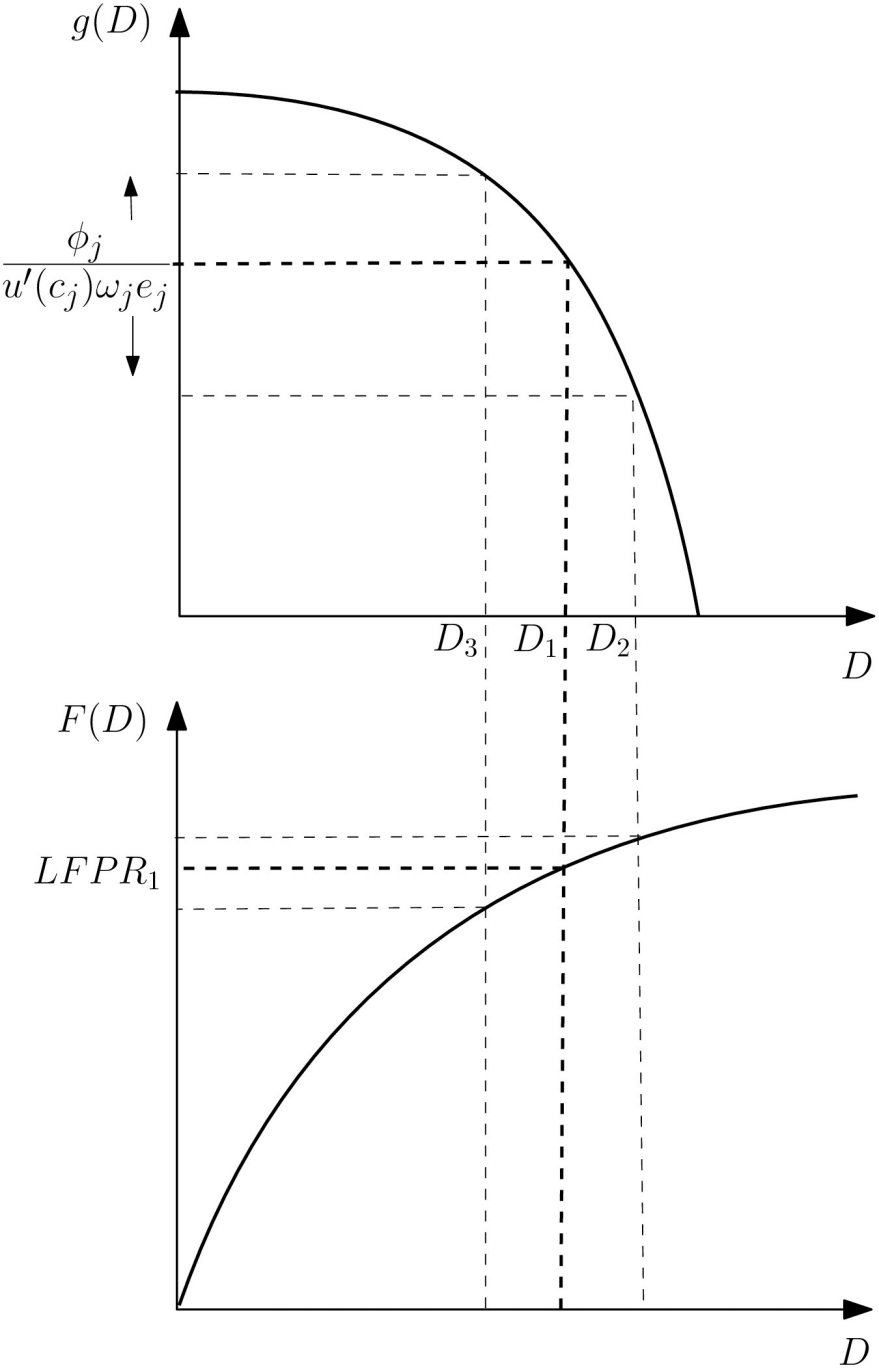
Health deficits and labor force participation. The upper panel shows the association between health deficits and productivity and the optimal deficit level at entry into retirement (the threshold). The lower panel shows the distribution of health deficits in a population and the implied rate of labor force participation.

The top panel of Fig 2 illustrates the participation constraint for labor supply. In the benchmark case, only individuals with fewer than *D*_1_ health deficits supply labor. The lower panel of Fig 2 shows the cumulative distribution function of health deficits for a population of given chronological age. The labor force participation rate (LFPR) can be read off directly from Fig 2 since only individuals with deficits below the *F*(*D*)–curve supply labor.

The participation constraint changes with advancing chronological age. As individuals gather more experience, *e*_*j*_(*t*) rises, and the threshold moves down (such that the critical deficit level moves up, for example, from *D*_1_ to *D*_2_). This feature reflects the fact that withdrawing from the labor force causes larger income losses due to experience as individuals grow older. In particular, for young and middle-aged workers this effect could be the dominating force such that labor supply increases with age. For the elderly, however, it is likely that the gains from experience have asymptotically reached its limit such that the physiological aging effect (strongly) dominates. In summary, the theory predicts a strong negative impact of health deficits on LFPR for the elderly while the effect is smaller for the young and middle aged. The reason is that, on average, elderly workers have developed more health deficits such that a greater share of them has frailty index in the range where workers consider retirement.

We can also use the theory to discuss the evolution of the LFPR of cohorts over time. The *F*(*D*)–curve of later-born cohorts is first order stochastically dominated by *F*(*D*) of earlier cohorts if later-born cohorts are healthier at any age (as the studies for European countries and the U.S., suggest [5, 6]. Furthermore, the LFPR may shift over time due to changing education or changing preferences for LFP (of, for example, women) as well as due to technological progress and income growth. With growing income, *ω*_*j*_ increases, shifting the threshold upwards. However, consumption increases as well, implying that *u*′(*c*) goes down and that the movement of the LFPR is ambiguous. For the special case of log-utility and a constant savings rate, the threshold stays constant under technological progress and income growth. Generally, however, the LFPR may shift for various non-health-related reasons from one cohort to the next, a feature that highlights the importance of a cohort-based analysis.

For our empirical analysis we approximate the *F*(*D*)–curve by a logarithmic function. Allowing the parameters of the labor supply model to be country -, gender-, age-, and cohort-specific, the theory thus predicts that

$$\begin{array}{c} LFPR_{cgit}=\beta \;\text{log}\;D_{cgit}+FE, \end{array}$$ (11)

in which subindex *c*, *g*, *i*, and *t* identifies the cohort, gender, country, and age of the considered individuals and the *FE* term captures fixed effects of cohort, gender, and country, as well as interactions of the these fixed effects with chronological age. This provides the structural model for our regression analysis. The theory predicts that *β* < 0, which our analysis outlined below will be empirically testing.

## 3. Data and sampling

### 3.1. Disease prevalence data

Data on disease prevalence rates for men and women by five year age-groups were taken from the GBD database [44], which covers the period 1990 to 2019. Keeping with conventions in the literature on health deficits, the youngest cohort included in our analysis is age 20 to 24 when sampled. In constructing the frailty index, we follow the criteria set out in [32] and we strictly follow [43, 62] in the selection of disease items. This leaves us with 32 aging-related health conditions, which are listed in the S1 Appendix. The prevalence rates of these diseases are aggregated into the frailty index as suggested by Eq (3).

**Fig 3:**
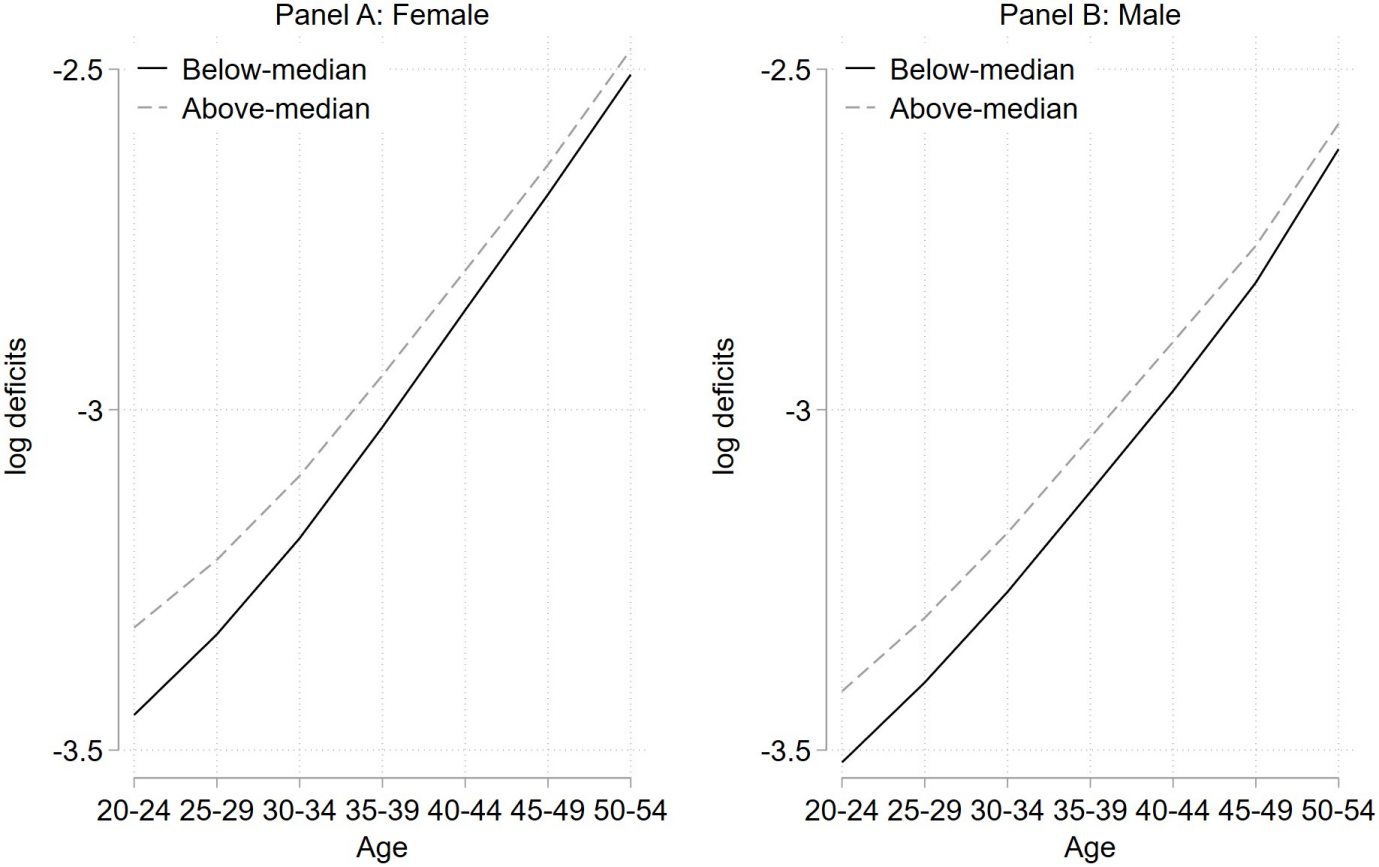
Compensation law of health deficits. **Notes**: This figure shows the development of the average frailty index over the life cycle from age 20–24 to 50–54. We compute four sub-samples: based on gender and above-below initial health deficits, measured at age 20–24. In each of these sub-samples, we calculate the average frailty index across countries and cohorts for each age-group. Panel A shows the age-group averages for females, while Panel B shows the age-group averages for males. We can only report these developments until age 50–54 since initial deficits (at age 20–24) are only observed for a sub-sample of our cohorts. See also Section 3.3.

Fig 3 provides empirical evidence for the compensating effect of deficits across countries. We split our sample of cohorts into above- and below-median initial deficits (measured at age 20–24) and for each age-group we compute average deficits across countries and cohorts. The estimates are reported for women in Panel A and men in Panel B. This shows that the compensating law of deficits is also present in our sample in that cohorts with different levels of initial deficits are converging in terms of deficits over the life cycle. The initial log level of deficits is inversely related to the growth rate of deficits, reflecting the compensation law of health deficit accumulation (see Section 2.2).

### 3.2 Labor supply data

**Fig 4:**
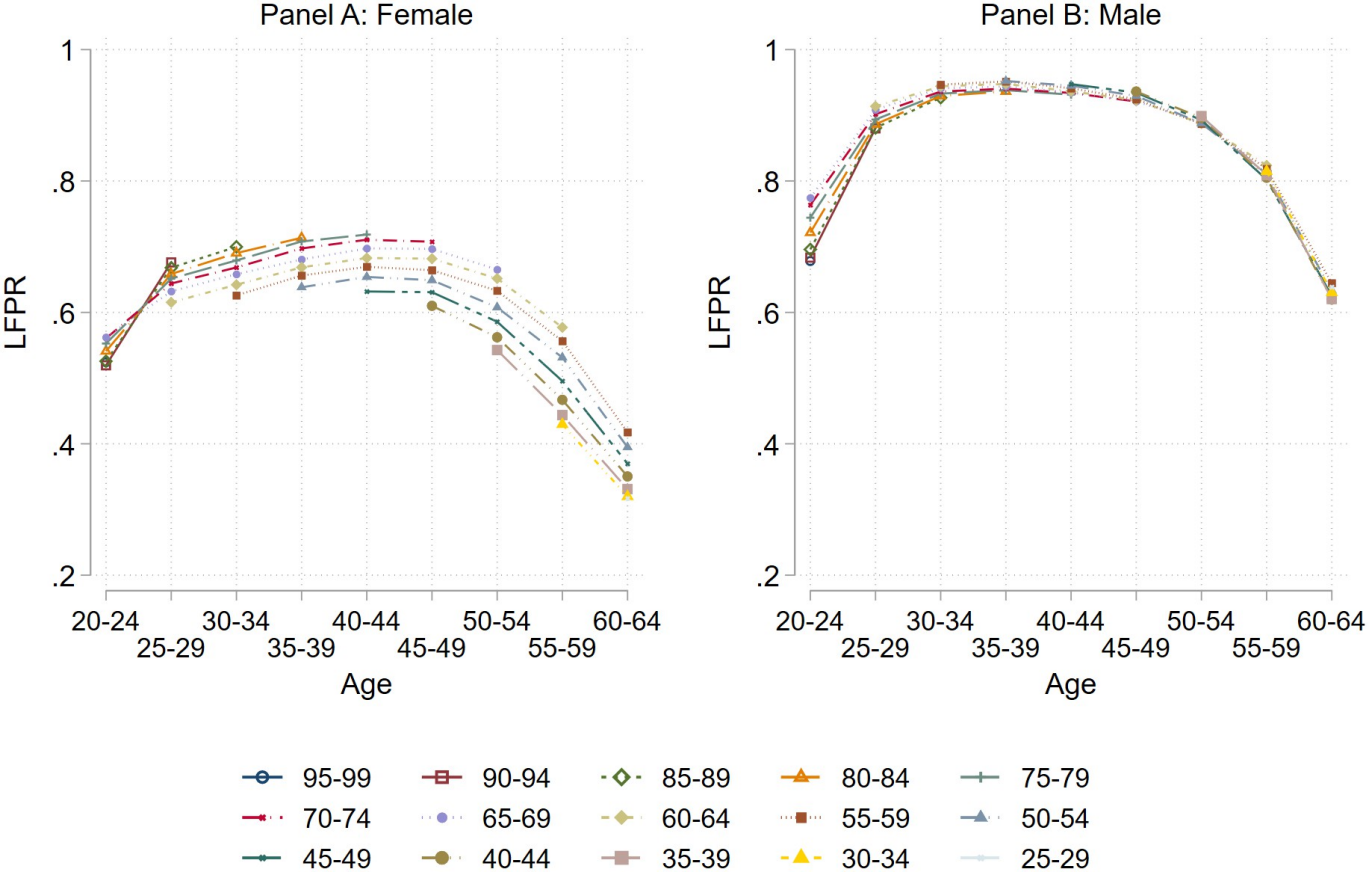
Labor force participation by gender and cohort. **Notes**: This figure shows the binned scatter plot of LFPRs from age 20 to 64 by gender and cohort. Before showing the LFPRs, we control for country fixed effects in the full sample. The sample here includes 15 different cohorts in 180 countries observed from 1990 to 2019. The numbers in the legend indicate birth years for the different cohorts; the oldest cohort being born from 1925 to 1929 (25–29), for example.

This subsection presents our data on life-cycle labor supply. Data on life-cycle labor market participation rates are drawn from the International Labour Organization’s (ILO) database [63]. We use ILO modeled estimates (November 2020), and the labor force participation rate is defined as the percent of the population working in given age, where working refers to both people who are employed and unemployed. Labor force participation rates (LFPRs) by gender, age, and country are, in principle, available annually at five-year age intervals. As our baseline sample consists of birth cohorts of five-year intervals observed from 1990 to 2019, we only need data on LFPRs (by gender, age, and country) every fifth year (1990, 1995,‥, 2019). (The latter age interval is only four years as we do not have data on deficits nor labor force participation from 2020 in the November 2020 ILO estimates.) The age groups available from the ILO database are: 20–24, 25–29, ‥, 60–64 and 65+, but since we use five-year birth cohorts, the latter age-group (65+) cannot be used in our analysis and is accordingly dropped. Fig 4 shows binned scatter plots of the LFPR over the life cycle by gender for our cohorts, while controlling for country fixed effects. For both men and women, we observe the well-known hump-shaped pattern of LFPRs in age. In addition, we see an upward movement of the labor supply curve for women (Panel A). After being merged together with our health deficits data, the dataset ends up consisting of 124 age-cohort-gender observations for 180 countries amounting to 22,320 observations in total. However, once we control for GDP per capita in our regression framework, the sample reduces to 164 countries.

### 3.3. Cohort sample structure

**Fig 5:**
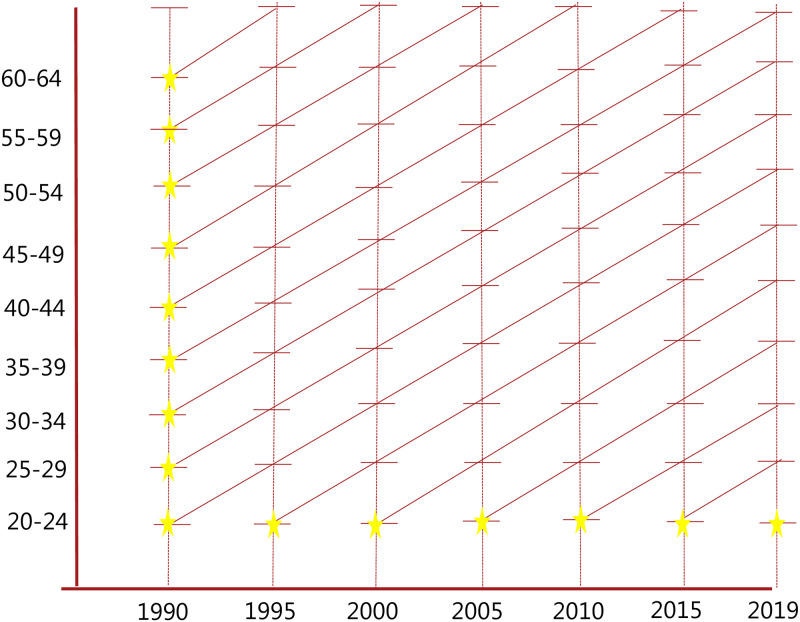
Lexis diagram for sampled cohorts from 1990 to 2019. **Notes**: This figure shows the cohort–year structure of our data. Each cohort is represented by a diagonal line. The yellow stars indicate the first observation for each cohort, i.e. the time and age when initial deficits are measured for a cohort. It is evident that our limited sample window prevents us from measuring initial deficits for all cohorts at age 20–24 and for these cohorts we instead measure them when the cohort enters sample window in 1990. For example, for the cohort aged 20–24 in 1990, initial deficits are observed at age 20–24 whereas for the cohort aged 25–29 in 1990, initial deficits are observed at age 25–29.

We consider five-year (birth) cohorts, which in our baseline sample, includes cohorts born: 1925–1929, 1930–1934, ‥, 1995–1998. As the Lexis diagram in Fig 5 illustrates, our data allow us to observe both physiological aging and labor participation for these cohorts from 1990 to 2019 up to the age category 60–64, corresponding to the so-called diagonal “life lines”. The yellow stars indicate when initial deficits are measured for the different cohorts. Because of data availability on deficits, we are not able to measure deficits at the beginning of the life cycle (age 20–24) for cohorts born before 1965, so initial deficits are measured at older ages for these cohorts. We account for this problem empirically by additionally controlling for age-cohort and age-country fixed effects in the 2SLS regressions. In addition, we cannot use the youngest cohort (born 1995–1998) and the oldest cohort (born 1925–1929) in the regressions, as we do not observe any age changes in deficits and labor force participation for these cohorts.

## 4. Estimation strategy

This section explains how we estimate the effect of physiological aging on labor supply. Motivated by our theoretical model for health deficits and labor supply, the structural equation takes on the following form:

$$\begin{array}{c} LFPR_{cgit}=\beta \;\text{log}\;D_{cgit}+\theta_{gt}+\delta_{cgi}+\varepsilon_{cgit}, \end{array}$$ (12)

where *LFPR*_*cgit*_ is the labor force participation rate of birth cohort *c* with gender *g*, living in country *i*, observed at age *t*. The corresponding frailty index (in natural logarithm) is given by log *D*_*cgit*_. As argued above, labor supply also likely depends on age, gender, cohort, and country-specific factors. In the OLS regressions of (12) we take these into account by including gender-age fixed effects (*θ*_*gt*_) and cohort-gender-country fixed effects (*δ*_*cgi*_). In the 2SLS estimation, we additional include country-age fixed effects (*θ*_*it*_) and cohort-age (*θ*_*ct*_) fixed effects in order to take into account that initial deficits are observed later in life for older cohorts, as explained above.

We estimate the coefficient of interest, *β*, by taking first differences in age, which differences out the cohort-gender-country fixed effect. This gives the following estimation equation:

$$\begin{array}{c} \Delta LFPR_{cgit}=\beta \Delta \;\text{log}\;D_{cgit}+\Delta \theta_{gt}+\Delta \varepsilon_{cgit}, \end{array}$$ (13)

where Δ*LFPR*_*cgit*_ is the change in the labor force participation rate from age *t* to *t* + 1, Δ log *D*_*cgit*_ is the change in log deficits (or the approximate growth rate), Δ*θ*_*gt*_ are gender-age fixed effects, and Δ*ε*_*cgit*_ is the error term, which is clustered at the country level.

Compared to the “level model” in Eq (12), this age-stacked, first-differences specification is easier to connect with our 2SLS strategy, which is going to exploit differences in initial deficits between cohorts as an instrumental variable for the growth rate in deficits as theoretically motivated by Eq (7). Based on this compensation law, the first stage is constructed as:

$$\begin{array}{c} \Delta \;\text{log}\;D_{cgit}=\alpha \;\text{log}\;D_{cgi}^{Initial}+\Delta {\tilde{\theta }}_{gt}+\Delta {\tilde{\varepsilon }}_{cgit}, \end{array}$$ (14)

where $\text{log}\;D_{cgi}^{Initial}$ is deficits measured at the beginning of the work-life cycle (age 20–24), which can only be measured for cohorts born later than 1965 given the GBD data on deficits. Therefore, we measure initial deficits in 1990 for cohorts born before 1965 when they enter our sample window (cf. Fig 5). In order not to compare older cohorts, where initial deficits are thus measured later in life, we follow two related approaches. First, we restrict the sample only to cohorts born after 1965, for which it is possible to measure initial deficit at age 20–24. Alternatively, we include all cohorts in the sample, but then control for cohort-age and country-age fixed effects in order not to make any misleading comparisons. Finally, note that the excluded instrument (initial deficits, $\text{log}\;D_{cgi}^{Initial}$) does not vary by age, and so our 2SLS estimation should essentially be thought of a number of stacked first differences (in age), and motivated by the law of compensating deficits, initial deficits are assumed to have the same impact on the change in logged deficits from one age to the next. Our identifying assumption, in terms of solving the inherent problem of reverse causality, is that labor market participation later in work life (e.g., at age 50–54) does not affect health deficits at beginning of the work life (age 20–24). In all reported regressions, we additionally control for logged GDP per capita, derived from Penn World Tables Version 10.01 [64], to capture changes in economic-wide conditions that could be correlated with labor market conditions and the frailty index.

## 5. Results

Table 1 reports the results from estimating different variants of Eq (13) by OLS. When not including any fixed effects, in column 1, we find that $\hat{\beta }=-1.5$ with a standard error of 0.075, implying that if deficits increase by one percent, labor force participation decreases by 1.5 percentage points. In the subsequent columns, when age and gender FE are included, we observed that the estimated effect reduces to about 0.25 to 0.27 percentage points. Including gender fixed effects in Eq (13) corresponds to controlling for gender-specific linear trends in Eq (12). Noting that health deficits increase, on average, by around 3 percent per year over the life cycle, even this magnitude implies a substantial drag on labor supply of physiological aging. The point estimate from specification (4) in Table 1 implies that a one-standard deviation of the health indicator (the standard deviation of the logged frailty index is 0.41) is associated with a decline in labor force participation by 10 percentage points. For comparison, using micro data, Vandenberghe estimated for a sample of workers aged 50–54 from 20 European countries that a one standard deviation in health is associated with a decline of labor force participation between 12 and 30 percentage points [18]. Our OLS estimates based on macro data are thus in line with estimates from the lower bound of a recent study using micro data.

**Table 1:** OLS estimates.

	(1) Δ*LFPR*	(2) Δ*LFPR*	(3) Δ*LFPR*	(4) Δ*LFPR*
Δ log *D*	-1.484***	-0.266***	-0.253***	-0.249***
	(0.075)	(0.050)	(0.050)	(0.056)
log *y*	-0.005***	-0.006***	-0.006***	-0.006****
	(0.001)	(0.001)	(0.001)	(0.001)
Observations	15,744	15,744	15,744	15,744
Age FE	No	Yes	Yes	Yes
Gender FE	No	No	Yes	Yes
Gender-age FE	No	No	No	Yes

**Notes**: This table reports OLS estimates of Eq (13). The dependent variable (Δ*LFPR*) is the change in labor force participation from one age to the next, while the main explanatory variable (Δ ln *D*) is the change in logged deficits. All regressions control for logged GDP per capita (log *y*), derived from from Penn World Tables Version 10.01 and estimated coefficients are reported in the table. Standard errors are robust and clustered at the country level.

*** p<0.01,

** p<0.05,

* p<0.1.

While the estimates reported in Table 1 hold constant a host of unobserved country-, age-, and cohort-factors, such as years of schooling, which is largely determined before entering work-life, they are unlikely to yield the causal effect of physiological aging on labor supply, since labor supply influences physiological aging (see the discussion of the related literature in the Introduction). For this reason, we expect the OLS coefficient of health deficits to be downward biased (in absolute value) and now turn our attention to the 2SLS strategy, which exploits the compensating law of health deficits accumulation to construct an instrumental variable.

**Fig 6:**
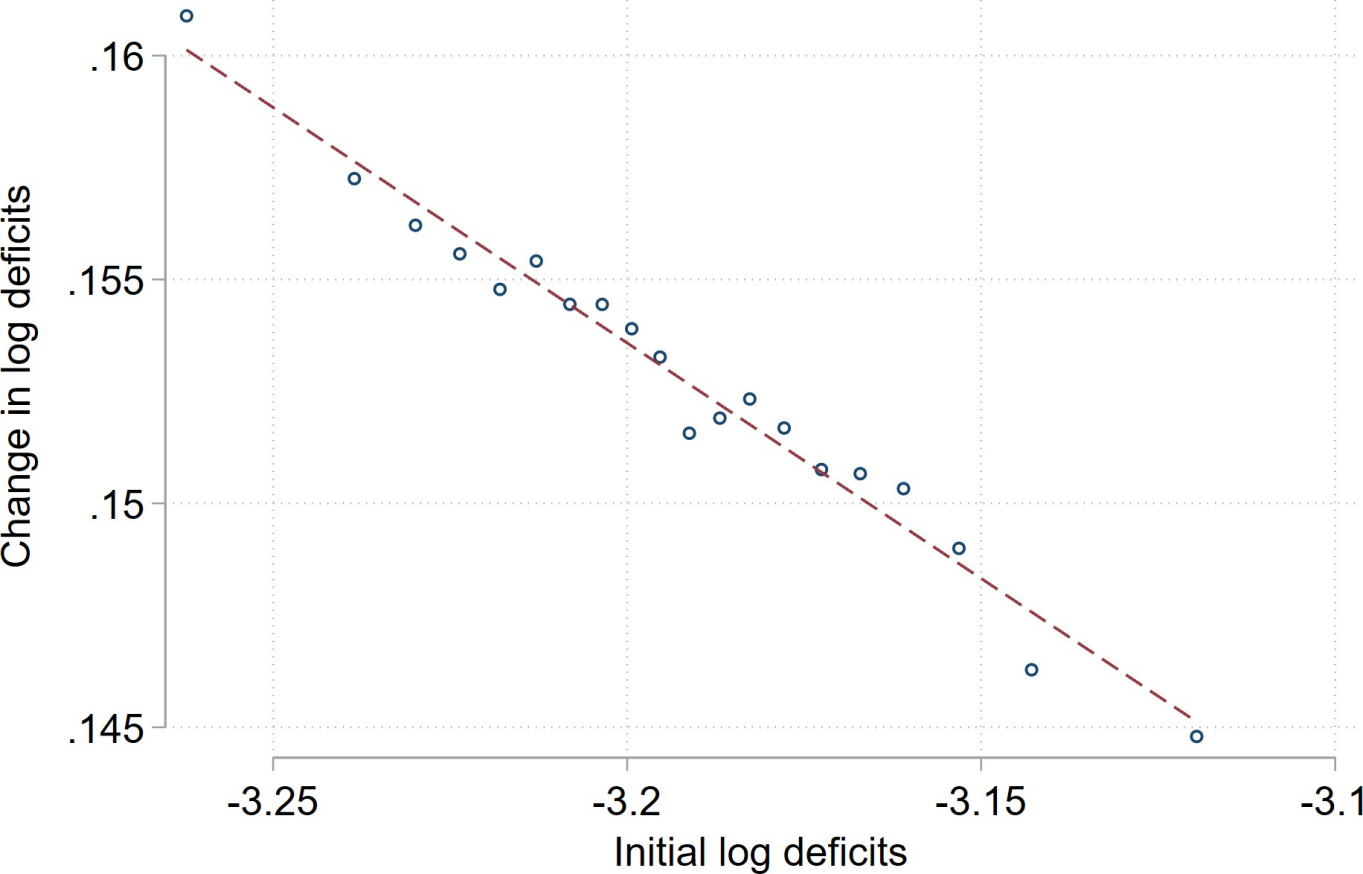
First-stage relationship between initial deficits and changes in deficits. **Notes**: The figure shows a binned scatter plot of the first-stage relationship, in which we include all cohorts and control for gender-age fixed effects, country-age fixed effects, and cohort-age fixed effects. $\hat{\alpha }=-0.11$ and standard error = 0.003.

In Fig 6, we depict the first-stage relationship as a binned scatter plot for our full sample of cohorts, while controlling for gender-age, country-age, cohort-age fixed effects, and logged GDP per capita. This first-stage coefficient is estimated as $\hat{\alpha }=-0.11$, which is statistically significant at the one percent level. This magnitude implies that if initial deficits decrease by a one standard-deviation of a natural log point (0.3), the growth rate of deficit increases by 3.6 percentage points. Given that we took differences of five year intervals, it implies a change of the annual growth rate of deficits by 0.72 percentage points. As can be seen from the bottom of Table 3, the Kleibergen-Paap F statistics ranges from 85 to 140 in all 2SLS specifications, indicating that the first-stage fit is strong, which reduces any concerns about weak-instrument biases.

Table 2 reports the resulting 2SLS estimates. In Columns 1–4, we include only cohorts born later than 1965, where (given our data on deficits) it is possible to measure initial deficits at the beginning of the work-life cycle. In these models, with the same fixed effects as in Table 1, the first-stage relationship is a little stronger compared to the model where all cohorts are included along with additional interaction fixed effects, reported in Fig 6 and specification (5) of Table 2. However, in all the reported models, the 2SLS estimates are in the range -0.7 to -0.4 (standard error circa 0.2), which is more than twice the numerical magnitude of the OLS estimates, reported in Columns 2–4 of Table 1. This pattern is consistent with the OLS estimate being downward biased due to reverse causality.

**Table 2:** 2SLS estimates.

	(1) Δ*LFPR*	(2) Δ*LFPR*	(3) Δ*LFPR*	(4) Δ*LFPR*	(5) Δ*LFPR*
Δ log *D*	-0.434*	-0.459*	-0.669***	-0.670***	-0.469**
	(0.257)	(0.249)	(0.203)	(0.203)	(0.190)
log *y*	-0.001	0.001	0.001	0.001	0.003
	(0.002)	(0.002)	(0.002)	(0.002)	(0.004)
Observations	6,888	6,888	6,888	6,888	15,744
Age FE	No	Yes	Yes	Yes	Yes
Gender FE	No	No	Yes	Yes	Yes
Gender-Age FE	No	No	No	Yes	Yes
Country-Age FE	No	No	No	No	Yes
Cohort-Age FE	No	No	No	No	Yes
Cohorts	>1965	>1965	>1965	>1965	all
First-stage F stat.	85.38	89.56	139.7	139.5	123.7

**Notes**: This table reports 2LS estimates of Eq 13, using Eq 14 as the first stage. The dependent variable (Δ*LFPR*) is the change in labor force participation from one age to the next, while the main explanatory variable (Δ ln *D*) is the change in logged deficits. In Columns 1–4 only cohorts born before 1965 are include, while in Column 5 all cohorts are included. All regressions control for logged GDP per capita (log *y*), derived from from Penn World Tables Version 10.01 and estimated coefficients are reported in the table. Standard errors are robust and clustered at the country level.

*** p<0.01,

** p<0.05,

* p<0.1.

In a simple model of simultaneity, and assuming no omitted variable biases, the pattern of the estimates OLS and 2SLS coefficients indicate that the effect of labor-market participation on deficits could be positive, but rather small in magnitude. In fact, following the procedure in [65], in which we use the 2SLS coefficient, reported in column 5 of Table 2, to partial out the response of labor-market participation to deficits and use that as an instrument for labor market participation, we estimate a 2SLS coefficient equal to 0.05 (standard error = 0.006). Thus, taken at face value, labor market participation increases deficits (i.e., physiological aging).

The estimate from specification (4) in Table 2 implies that a one-standard deviation of the health indicator is associated with a decline in labor force participation by 27 percentage points. This estimate is in line with the upper bound of estimates from micro data [18].

### 5.1. Age and labor force participation: Simulations

**Fig 7:**
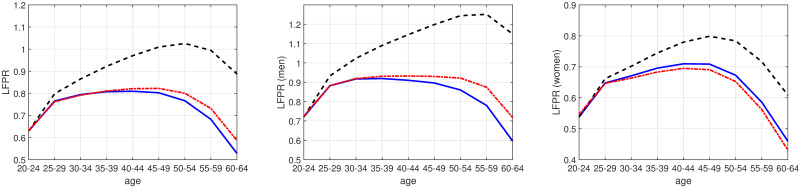
Age and labor force participation: Simulations. Blue (solid) lines: predictions when health deficits are omitted as explanatory variable. Red (dashed) lines: predictions when log deficits are included as regressor and deficits increase by 2.9 percent per year. Black (dash-dotted) line: predictions when log deficits are included as regressor but deficits are held constant for prediction.

In this section, we aim to assess the explanatory power of physiological aging for labor force participation rates (LFPRs) by way of simulation. The simulations are based on the level estimates shown in Table 3 and the evolution of health deficits by age. We first consider labor supply of an average world citizen in the panel on the left-hand side of Fig 7. The blue (solid) line shows the age-coefficients when the LFPR is regressed on age fixed effects and gender-country-sex fixed effects (reported in column 1 of Table 3). According to this view, increasing as well as declining LFPRs is “explained” by chronological age. This model suggest that labor supply starts declining from about age 40 because workers are getting older. It could have been derived from a standard Mincer model, according to which worker productivity and wages decline with chronological age as elderly workers grow older.

**Table 3:** Level estimates.

	(1) *LFPR*	(2) *LFPR*	(3) *LFPR*	(4) *LFPR*	(5) *LFPR*	(6) *LFPR*
ln D		-0.295***		-0.124		-0.445***
		(0.055)		(0.079)		(0.076)
age 25–29	0.136***	0.169***	0.110***	0.124***	0.161***	0.212***
	(0.005)	(0.008)	(0.006)	(0.010)	(0.006)	(0.011)
age 30–34	0.165***	0.236***	0.133***	0.164***	0.196***	0.303***
	(0.006)	(0.015)	(0.007)	(0.020)	(0.007)	(0.021)
age 35–39	0.178***	0.293***	0.158***	0.207***	0.198***	0.369***
	(0.007)	(0.023)	(0.008)	(0.032)	(0.008)	(0.032)
age 40–44	0.180***	0.341***	0.172***	0.242***	0.189***	0.425***
	(0.007)	(0.031)	(0.008)	(0.045)	(0.008)	(0.042)
age 45–49	0.173***	0.380***	0.171***	0.261***	0.175***	0.477***
	(0.007)	(0.039)	(0.009)	(0.057)	(0.008)	(0.053)
age 50–54	0.137***	0.396***	0.135***	0.246***	0.139***	0.523***
	(0.008)	(0.049)	(0.009)	(0.070)	(0.008)	(0.067)
age 55–59	0.053***	0.365***	0.048***	0.179**	0.059***	0.530***
	(0.008)	(0.058)	(0.010)	(0.082)	(0.010)	(0.081)
age 60–64	-0.101***	0.258***	-0.078***	0.071	-0.125***	0.428***
	(0.013)	(0.067)	(0.014)	(0.093)	(0.015)	(0.095)
Observations	20,008	20,008	10,004	10,004	10,004	10,004
Cohort-country-sex FE	Yes	Yes	Yes	Yes	Yes	Yes
Sample	All	All	Female	Female	Male	Male

**Notes**: This table reports OLS estimates of Eq 12. The dependent variable (*LFPR*) is the labor force participation rate, while the explanatory variable (log *D*) is logged deficits. Age 20–24 is the reference age group for the age dummies. columns 1 and 2 include only all cohorts, while Columns 3–6 split the sample by gender, which means that these specifications only absorb country-cohort fixed effects. Standard errors are robust and clustered at the country level.

*** p<0.01,

** p<0.05,

* p<0.1

The red (dashed) line shows the predicted LFPR by age when logged deficits are added in the regression and age-group-specific health deficit are fed into the model. Estimates are taken from column 2 of Table 3. For these figures we estimated the association between age and the log of health deficits as in [43]. We found that new health deficits are accumulated at a rate of 3.1 percent per year of age, very close to the estimate of 2.9 percent per year of age in [43]. This average speed of physiological aging implies that the frailty index increases from about 0.03 at age 20–24 to 0.10 at age 60–64 (see also Fig 3). The predicted LPFR by age closely traces the age-specific LFPR but the explanation differs. Now, deteriorating health is the main driver of declining LFPRs. This can be seen by the black (dash-dotted line), which reports predicted LFP assuming constant health. Controlling for health status, labor force participation is predicted to increase until age 50–54 and the LPFR at age 60–64 is as high as at age 30–24. The area between the red and the black line provides an estimate of the increase in LFPR that could be achieved by abolishing physiological aging. The estimates suggest a gain in LFPR of about 20 percent at age 44–49 and of about 30 percent at age 60–64.

The middle panel of Fig 7 shows results when the simulation exercise is repeated in a sample restricted to men (estimates from columns 5 and 6 of Table 3). The predictions with and without consideration of physiological aging (the red and blue line) trace the actual LFP quite well, as a comparison with Fig 4 show. The black (dash-dotted) line, showing LFP with constant health, reveals that the potential LFP gain from slowing down physiological aging is even greater than in the full sample. Of course, an LFPR above 1 makes no sense. Assuming an upper boundary of potential LFP at or below 1, the estimates thus suggest that there is no reduction in LFPR due to chronological aging.

The panel on the right-hand side of Fig 7 shows the estimated and simulated results for women. Again, the predictions trace the actual LFPR quite well (cf. Fig 4). The estimated coefficient of log deficits is smaller for women and therefore the impact of deteriorating health on labor supply is also smaller. The potential gain in LFP from abolishing physiological aging, however, is still substantial. It is about 10 percentage points at age 44–49 and almost 20 percentage points at age 60–64. If we assume an upper bound for LFP at or below 0.7, for example because of pregnancy and child rearing, we would arrive at a similar conclusion as for men: advancing chronological age contributes little to the decline in the LFPR when physiological age is held constant.

## 6. Conclusion

In this paper, we contributed to the literature on the labor-market participation effects of health with a novel approach using macro data. Instead of using individuals as subjects of investigation, we considered cohorts born between 1925 and 1995 from 180 countries and estimated the impact of deteriorating health on labor force participation. Drawing on research in the fields of biology and medicine and aging-related health data on prevalence rates of health deficits from the GBD study, we computed the frailty index as a measure of physiological aging over the life cycle. The panel structure of the data over 1990–2019 allowed us to follow cohorts over time and to control for a host of potential confounders by country, gender, age, and cohort fixed effects. We exploited the association between initial health deficits and the growth rate of health deficits (the compensation law of morbidity) as an instrumental variable to reduce the problem of reverse causality.

The results suggest a strong negative effect of physiological aging on labor market participation. According to the OLS estimates, a one standard deviation increase of deficits is associated with a decline in labor force participation by about 12 percentage points, an estimate that aligns with the lower bound of recent estimates from European micro data. In 2SLS regressions, we find a substantially larger effect, according to which an increase in the frailty index by one percent is associated with an 0.6 percentage point decline of the labor force participation rate. This means that an increase of deficits by one standard deviation leads to a decrease of labor force participation by 30 percentage points, an estimate that aligns with the upper bound of recent micro estimates. Simulations, in which we feed the actual (average) increase of health deficits into an estimated model of life cycle labor supply, we find that almost all decline in labor supply of the elderly can be motivated by deteriorating health and that advancing chronological age (i.e. experience) exerts a positive impact on labor supply until age 55.

The main policy conclusion is thus that, if health of the workforce could be improved, population aging would much less of a concern for labor force participation rates of the population below age 65. Historical studies by Costa show that the deterioration of the human body slowed down since the beginning of the 20th century such that later born cohorts of elderly US American men (50–64 years old) experience less impairments of bodily function [66, 67]. Costa shows that a host of health conditions improved quite strongly, some, such as joint problems, back problems, and heart and circulatory conditions, improved at a rate more than twice as fast as the improvement of life expectancy. In a sample of European countries as well as in the US, average health deficits at any age above 50 declined by 1.0–1.4 percent per year of later birth [6, 68]. These observations suggest that productivity and labor force participation, in particular of elderly individuals, could be stimulated by improving health.

These trends, however, are not yet visible at the global level. Inspection of our GBD-based data reveals that that younger cohorts in non-western and poor countries are less healthy at all working ages [43]. Physiological aging thus operates against the “demographic dividend” that could be derived from the relatively young work force in these countries [69]. Later born workers in western and rich countries, in contrast, do not experience these negative trends and benefit from (mildly) improved health, in particular at later working ages, in line with the results from the micro studies cited above. These trends, however, are not (yet) sufficiently strong to offset the deleterious effects of population aging on labor supply.

## Supporting information

S1 Appendix (PDF)
