# Supplementary material for: Physiological aging and life-cycle labor supply across countries
Source: PLoS One. 2023 Nov 29;18(11):e0294952. doi: 10.1371/journal.pone.0294952 (PMC10686504; doi:10.1371/journal.pone.0294952)
Supplement: S1 Appendix — (PDF) [file pone.0294952.s001.pdf]

Physiological Aging and Life-Cycle Labor Supply across Countries

Appendix

Casper Worm Hansen[1]
Carl-Johan Dalgaard[1]
Holger Strulik[2]

[1] Department of Economics, University of Copenhagen, Øster Farimagsgade 5, 1353 København, Denmark.
[2] University of Göttingen, Department of Economics, Platz der Göttinger Sieben 3, 37073 Göttingen, Germany; Correspondence to holger.strulik@wiwi.uni-goettingen.de.

A. Items in the Frailty Index

The frailty index is based on prevalence rates for the following diseases (32 in total):

Diarrheal diseases; Protein-energy malnutrition; Neoplasms; Ischemic heart disease; Stroke; Non-rheumatic valvular heart disease; Cardiomyopathy and myocarditis; Atrial fibrillation and flutter; Peripheral artery disease; Other cardiovascular and circulatory diseases; Chronic respiratory diseases; Peptic ulcer disease; Gallbladder and biliary diseases; Alzheimer's disease and other dementias; Parkinson's disease; Depressive disorders; Diabetes mellitus; Chronic kidney disease; Skin and subcutaneous diseases; Other sense organ diseases; Rheumatoid arthritis; Osteoarthritis; Low back pain; Gout; Urinary diseases and male infertility; Genital prolapse; Endocrine, metabolic, blood, and immune disorders; Oral disorders; Falls; Hearing loss; Heart failure; Blindness and vision loss.
